# Ileocolic Thrombophlebitis and Lymphadenitis Mimicking Acute Appendicitis as a Late Manifestation in a COVID-19 Patient: A Case Report

**DOI:** 10.7759/cureus.29019

**Published:** 2022-09-11

**Authors:** Meng-Yi Chou, Che-Yuan Cheng, Shing-Jhong Long, Kai-Wen Yang, Yung Hsu

**Affiliations:** 1 Radiology, Miaoli General Hospital, Ministry of Health and Welfare, Miaoli City, TWN; 2 Surgery, Miaoli General Hospital, Ministry of Health and Welfare, Miaoli City, TWN

**Keywords:** covid-19, lymphadenitis, appendicitis, thrombophlebitis, ileocolic branch, mesenteric venous thrombosis

## Abstract

Coronavirus disease 2019 (COVID-19) is an infectious viral disease, manifesting primarily as a lung infection with fever and respiratory symptoms. However, it also has a wide range of gastrointestinal symptoms, including nausea, vomiting, abdominal pain, and diarrhea. Right lower quadrant (RLQ) abdominal pain is a common complaint for patients seeking care at emergency departments. In addition to appendicitis, the other possible causes include diverticular disease, epiploic appendagitis, Crohn’s disease, or mesenteric lymphadenitis, among others. Mesenteric ischemia is an uncommon, but crucial cause of abdominal pain, necessitating early diagnosis and treatment. Herein, we report a 47-year-old man who presented to our emergency department complaining of RLQ abdominal pain following recovery from COVID-19. CT was performed due to concern for acute appendicitis. However, mesenteric thrombophlebitis and lymphadenitis in the ileocolic branch were noted on CT. His abdominal pain improved after receiving anticoagulation therapy. This case describes an uncommon etiology of RLQ abdominal pain that should be considered as a late complication of COVID-19.

## Introduction

Mesenteric vein thrombosis (MVT) is an uncommon but crucial cause of mesenteric ischemia. Early diagnosis of MVT relies on high clinical suspicion, especially in patients with hypercoagulable states, recent abdominal surgery, intra-abdominal infection, or portal hypertension [1]. The well-known predisposing factors of hypercoagulable states include malignancy, polycythemia vera, protein C deficiency, protein S deficiency, antithrombin III deficiency and factor V Leiden mutation, presence of lupus anticoagulant, and antiphospholipid syndrome [2]. Given the widespread outbreak of COVID-19 infection, increasing evidence shows that COVID-19 may be associated with hemostasis impairment, which predisposes patients to both venous and arterial thromboembolism [3].

Currently, COVID-19-related coagulopathy is another important cause of venous thrombosis. A meta-analysis of previous studies reported that deep vein thrombosis (DVT) had an overall prevalence of 11.2% and pulmonary embolism (PE) 7.8% in those needing hospitalization [4]. Only case reports or series have shown portal vein thrombosis, mesenteric vein thrombosis, and Budd-Chiari syndrome as complications of COVID-19 [3]. To date, isolated thrombophlebitis of the ileocolic branch of superior mesenteric vein has not been reported and only one case was reported with mesenteric lymphadenitis as an atypical presentation of COVID-19 [5]. Here we report a specific case of mesenteric thrombophlebitis and lymphadenitis in the ileocolic branch that mimicked acute appendicitis in the clinical presentation of right lower quadrant (RLQ) abdominal pain as a late complication in a COVID-19 patient.

## Case presentation

**Figure 1:**
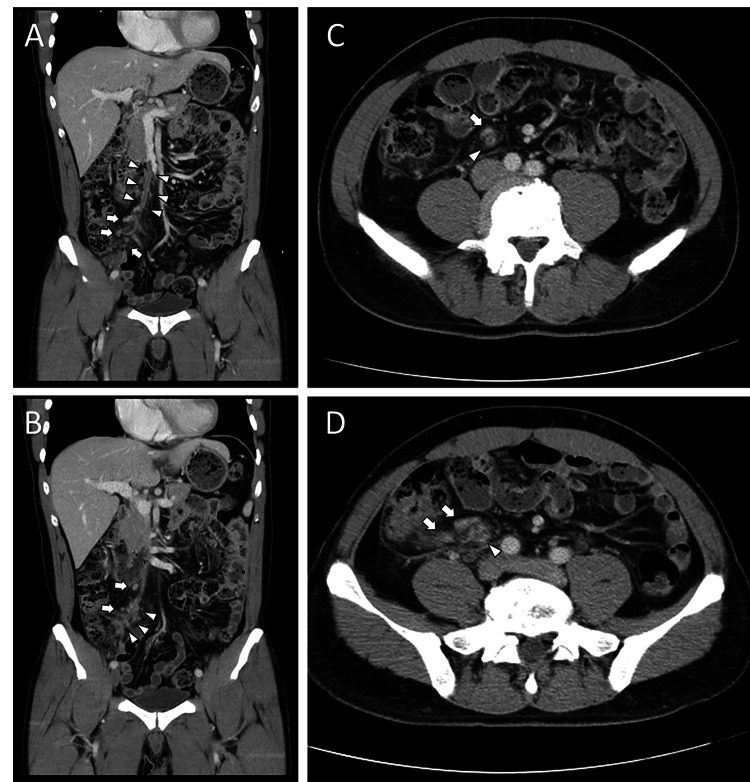
Contrast-enhanced abdominal CT scan Coronal plane (A and B) and axial plane (C and D) of contrast-enhanced CT revealed thrombus (white arrowheads) in the ileocolic branch of superior mesenteric vein with increased regional lymphadenopathy (white arrows) and fat strandings.

A 47-year-old male patient presented to our emergency department with abdominal pain for 10 days. He had COVID-19 infection with mild upper respiratory tract symptoms three weeks prior but had recovered after conservative treatment. Besides the recent history of COVID-19, he was relatively healthy and had received three doses of the COVID-19 vaccine. He had been to a local clinic and had taken oral antibiotics for abdominal pain. However, he came to our outpatient department due to persistent abdominal pain, and fever had also developed. He had no coughing, sore throat, dyspnea, chest pain, nausea, vomiting, or diarrhea. He was referred to our emergency department under the tentative diagnosis of appendicitis because the physical examination revealed moderate tenderness at RLQ abdomen without muscle guarding or rebounding tenderness. Laboratory data revealed elevated white blood count of 18.63x103/uL (reference value: 4-10.8x103/uL), C-reactive protein of 9.36 mg/dL (reference value: up to 0.29 mg/dL), and D-dimer levels of 10.23 mg/L fibrinogen equivalent units (FEU; reference value: up to 0.55 mg/L FEU). The COVID-19 reverse transcription-polymerase chain reaction (RT-PCR) test was weakly positive with a cycle threshold (Ct) value of 33.35. Other laboratory data were unremarkable. His chest X-ray revealed no active lesion in bilateral lung fields. Multi-slice CT of the abdomen and pelvis with and without intravenous contrast enhancement was performed for suspicion of acute appendicitis, diverticulitis, or COVID-19-induced colitis. However, the CT revealed segmental filling defect within the ileocolic branch of superior mesenteric vein with increased regional lymphadenopathy and fat strandings, without evidence of appendicitis, diverticulitis, or colitis (Figure 1).

With the diagnosis of mesenteric thrombophlebitis and lymphadenitis in the ileocolic branch, he was admitted and received systemic anticoagulant therapy with enoxaparin 100mg Q12H, and the antibiotics with piperacillin 4000mg/tazobactam 500mg Q8H. Subsequent laboratory data revealed protein C of 95.6% (reference value: 70% to 140%), protein S activity of 129.5% (reference value: 60% to 130%) and was negative for antiphospholipid antibodies (APA). After five days of treatment, his fever and abdominal pain gradually subsided. He was then discharged with oral edoxaban 60mg QD and amoxicillin 875mg/clavulanic acid 125mg BID. He was still free of symptoms during follow-up at our outpatient department.

## Discussion

Severe acute respiratory syndrome coronavirus 2 (SARS-CoV-2) has been causing COVID-19 illness globally since December 2019. SARS-CoV-2 targets the lungs and other organs of the body via angiotensin-converting enzyme 2 (ACE2) and the serine protease transmembrane protease serine 2 (TMPRSS2) receptors for cell entry [6]. Because the ACE2 receptor is highly expressed throughout the gastrointestinal tract, SARS-CoV-2 may enter gastrointestinal cells via ACE2 receptors to cause direct damage to the gastrointestinal organs [7,8]. SARS-CoV-2 also causes endothelial injury of the splanchnic vein via ACE2 receptor and hypercoagulability due to increased inflammatory response [3,9]. In critically ill patients, immobilization further predisposes an individual to the development of venous thrombosis due to venous stasis [9,10]

The incidence of thromboembolic events in hospitalized COVID-19 patients was reported to be 5.3% in a Japanese study, which was up to 13% of patients with severe respiratory conditions. Among those patients, 48% of events were arterial thrombosis and 52% were venous thrombotic events. Pulmonary thrombosis and deep vein thrombosis account for most cases of venous thrombosis [11]. According to a recent review of acute splanchnic vein thrombosis in patients with COVID-19, portal vein thrombosis was most frequently reported, followed by mesenteric vein thrombosis, splenic vein thrombosis, and then Budd-Chiari syndrome [3]. Two case reports describe subacute mesenteric venous thrombosis that developed one week after COVID-19 diagnosis [12,13]. Isolated thrombophlebitis in the ileocolic branch of the superior mesenteric vein is exceptionally rare and has been reported as a complication of acute appendicitis [14]. In the present case, the patient developed isolated thrombophlebitis in the ileocolic branch of the superior mesenteric vein, without evidence of acute appendicitis or diverticulitis, following recovery from COVID-19. Patell et al. also reported the cumulative incidence of 2.5% for arterial and venous thrombosis events at day 30 and the cumulative incidence of venous thromboembolism alone at day 30 was 0.6% following hospital discharge for COVID-19 [15].

The most common symptom of mesenteric vein thrombosis is abdominal pain, followed by vomiting and fever [3]. In the present case, the abdominal pain was more severe in the RLQ when thrombosis was only in the ileocolic branch of the superior mesenteric vein. Patients experiencing thrombotic events usually have higher white blood cell counts, C-reactive protein (CRP), lactate dehydrogenase (LDH), ferritin, D-dimer, fibrinogen, and fibrinogen degradation products (FDP), prolonged prothrombin time (PT), and lower lymphocyte counts and hemoglobin levels than those without thrombotic events. Of note, elevated D-dimer and ferritin levels are independent risk factors for thrombosis [11]. Multi-phase CT is the modality of choice for the diagnosis of mesenteric venous thrombosis. Filling defects in the superior mesenteric vein or its branches is suggestive of thrombosis. The association with lymphadenitis, bowel changes, or pneumatosis intestinalis should also be evaluated. In addition, CT may be used to exclude other etiologies, such as mesenteric arterial occlusion, acute appendicitis, diverticulitis, or others [1,16,17].

Although small bowel necrosis occurs less often in mesenteric vein thrombosis due to preserved arterial supply and venous drainage via collaterals, eventually intestinal infarct ensues as ischemia progresses [16]. Low-molecular-weight heparin (LMWH) is currently recommended as the first-line treatment for in-hospital patients with COVID-19 requiring anticoagulation [18]. Surgery is usually reserved for patients with intestinal infarction, and endovascular thrombolysis/thrombectomy is a viable option that can be considered in patients without evidence of bowel necrosis [19]. In the present case, the patient’s symptoms subsided after five days of anticoagulation with LMWH and he was shifted to edoxaban after discharge.

## Conclusions

This case describes a late COVID-19 thrombotic manifestation in the ileocolic branch of the superior mesenteric vein with clinical symptoms mimicking acute appendicitis. Elevated D-dimer may be a clue to mesenteric venous thrombosis in patients who developed abdominal pain following recovery from COVID-19. Multi-phase CT is the modality of choice for the definitive diagnosis. Earlier diagnosis of mesenteric venous thrombosis is of great importance to ensure earlier treatment of the patient with anticoagulation.
